# Comparison of Associations, Outcomes, and Drug Susceptibility Between Candida auris and Non-Candida auris Bloodstream Infections in a University Hospital in Muscat, Oman: A Retrospective Observational Study

**DOI:** 10.7759/cureus.113259

**Published:** 2026-07-23

**Authors:** Divya Deodhar, Prashanth K Prabhakar, Abjad Al Busaidi, Badriya Al Adawi, Abdullah Balkhair

**Affiliations:** 1 Department of Medicine, Infectious Diseases Unit, Sultan Qaboos University Hospital, Muscat, OMN; 2 Department of Clinical Microbiology and Immunology, Sultan Qaboos University Hospital, Muscat, OMN

**Keywords:** associations, bloodstream infection, candida auris, comparison, drug susceptibility, non-candida auris

## Abstract

The objective of this study was to compare the associations, outcomes, and drug susceptibilities of patients with *Candida auris* bloodstream infection with those of patients with non-*C. auris* bloodstream infections in Muscat, Oman. A retrospective observational study was conducted that included all adult patients (≥18 years) with *Candida* bloodstream infection over a five-year period, from January 2018 to December 2022. A total of 160 patients were identified during the study period and constituted the study sample. They were divided into two groups: the *C. auris* group, comprising 58 patients, and the non-*C. auris* group, comprising 102 patients. On comparing the two groups, prior colonization with *Candida* species was one of the strongest associations with *C. auris* bloodstream infection, with an OR of 6.71 (95% CI, 3.26-13.80; p < 0.00001). Similarly, previous health care contact within the last 90 days was significantly associated with *C. auris* bloodstream infection (OR, 5.89; 95% CI, 2.61-14.24; p < 0.00001). Echinocandins may be considered an empiric treatment option in patients with suspected *Candida* bloodstream infection, particularly those with these associations, to target *C. auris* in this region.

## Introduction

Bloodstream infection with *Candida* is a serious invasive fungal infection. It is considered the third most common cause of bloodstream infections in ICUs in the United States [1]. The incidence of health care-associated candidemia has risen by over 50% in the last decade [2]. Factors contributing to this increase include the presence of central venous catheters (CVCs), patient frailty, immunosuppression, overuse of antibiotics, delayed diagnosis, and variability in antifungal susceptibility. Other risk factors, such as hematological malignancies, total parenteral nutrition (TPN), and major abdominal surgery, also predispose patients to candidemia [3]. Candidemia has contributed to poor patient outcomes, longer hospital stays, substantial health care costs, morbidity, and mortality [2]. Mortality rates vary, with estimates ranging from 30% to 60%. Some studies indicate that overall mortality can be as high as 40.4% [4].

Multiple species of *Candida* have been found to cause human disease, but more than 90% of invasive disease is caused by the five most common pathogens: *Candida albicans*, *Candida glabrata*, *Candida tropicalis*, *Candida parapsilosis,* and *Candida krusei *[5]. This proportion may vary depending on the geographical region and patient population. Overall, *C. albicans *remains the leading cause of candidemia. Non-albicans *Candida *species account for approximately two-thirds of cases [6]. There has been a major shift in the *Candida* species causing invasive bloodstream infections. However, *C. albicans* remains the most common species [7]. Non-albicans *Candida *species, such as *C. glabrata*, *C. parapsilosis*, and *C. tropicalis*, have been found to exhibit reduced susceptibility or intrinsic resistance to antifungal agents, particularly fluconazole, which can lead to persistent infection and increased mortality [8].

Since its first isolation from the ear canal of a 70-year-old Japanese woman in Tokyo in 2009, *Candida auris* has spread rapidly and has been reported worldwide [9,10]. The risk factors associated with *C. auris *infection are similar to those for other *Candida* species and include ICU stay, CVC, broad-spectrum antibiotic use, and prior antifungal use. However, differences in antifungal susceptibility pose a major challenge in its treatment. Most* Candida *species cause infection through autoinfection from the host's endogenous microflora. However, *C. auris *is primarily acquired exogenously and is likely transmitted through contaminated environments, equipment, medical devices, and person-to-person contact via contaminated skin. Its ability to survive on biotic and abiotic surfaces for several weeks or even months makes it one of the most persistent organisms in the hospital environment. Therefore, implementation of strict infection prevention and control practices is essential to prevent transmission and subsequent invasive infections within health care settings, as *C. auris *is associated with cross-transmission, drug resistance, prolonged hospitalization, increased health care costs, morbidity, and high mortality [11].

Because of its high rate of antifungal resistance, *C. auris *is more difficult to treat, requires longer hospital stays, and is associated with higher morbidity and mortality than other *Candida *species. Approximately 90% of *C. auris *isolates in the United States are reported to be resistant to fluconazole, and 30% are resistant to amphotericin B, with resistance rates continuing to increase [12]. Resistance in *C. auris* is expanding, and echinocandin-resistant *C. auris* has also been reported [13]. Because echinocandins are the first-line therapy for invasive *Candida* infections caused by *C. auris*, resistance may make these bloodstream infections difficult to treat [14]. In a systematic review by Kim et al., the reported 30-day mortality rates associated with *C. auris *candidemia ranged from 29% to 62% across different studies [11].

There has been a significant increase in *C. auris* cases in Oman since it was first reported in 2016, with outbreaks occurring in health care institutions throughout the country between 2018 and 2019 [15,16]. Despite its rising incidence and significant health care impact in the country, there is a lack of studies comprehensively comparing *C. auris *infections with non-*C. auris *infections. Therefore, this study aimed to compare the clinical associations, in-hospital mortality, and antifungal susceptibility patterns between the two groups.

## Materials and methods

This retrospective observational cohort study was conducted at Sultan Qaboos University Hospital over a five-year period from January 2018 to December 2022. All adult patients with *Candida* bloodstream infection during the study period were included. Patients were identified from the electronic records of the microbiology laboratory through the Hospital Information System. All episodes of bloodstream infection caused by *Candida *species during the study period were included. Demographic, clinical, and microbiological data were collected from the electronic patient records using a predesigned pro forma. Identification of *Candida *species in the laboratory was performed using matrix-assisted laser desorption ionization-time-of-flight mass spectrometry (Bruker Corporation, Billerica, MA, USA). Antifungal susceptibility testing was performed using Sensititre YeastOne (Trek Diagnostic Systems Ltd, East Grinstead, UK). The obtained minimum inhibitory concentrations (MICs) were interpreted based on the CDC tentative breakpoints for *C. auris *[17].

The inclusion criteria were all adult patients (18 years or older) with a first episode of bloodstream infection caused by *Candida* species during the study period and all hospitalized patients with bloodstream infection caused by a single *Candida *species during the study period. The exclusion criteria were patients with more than one episode of *Candida *bloodstream infection during the study period, for whom subsequent episodes were excluded; patients with bloodstream infections caused by dual *Candida *species during the study period; and patients with missing data in the Hospital Information System.

The study cohort comprised 182 episodes of bloodstream infection caused by *Candida *species. Of these, 12 repeat admissions during the study period, seven episodes of dual-species *Candida *bloodstream infection, and three patients with missing data in the electronic patient record were excluded. A total of 160 patients constituted the study cohort. These patients were divided into two groups to achieve the objectives of the study. The first group comprised patients with *C. auris *bloodstream infection (the *C. auris *group). The second group, referred to as the non-*C. auris *group, included all patients with bloodstream infections caused by other *Candida *species. The study was approved by the Hospital Medical Research Ethics Committee (reference number SQU-EC/116/2023; MREC #2997).

The collected data were entered into a Microsoft Excel spreadsheet (Microsoft Corporation, Redmond, WA, USA). Descriptive statistical methods, including medians and percentages, were used where appropriate. Comparisons between the two groups were performed using IBM SPSS Statistics for Windows, version 22.0 (released 2013; IBM Corp., Armonk, NY, USA) for data analysis. A p-value of <0.05 was considered statistically significant.

The definitions used in the study were as follows. Time to positivity was defined as the number of days from hospitalization to the first positive blood culture for *Candida *species. Previous health care contact was defined as any contact with the health care system within the previous 90 days. Prior colonization was defined as a positive culture for *Candida *species from urine or from rectal and nasal swabs obtained for routine screening for methicillin-resistant *Staphylococcus aureus* and multidrug-resistant organisms, according to hospital protocol, or a positive culture for *Candida *species from urine, respiratory specimens, or sterile body fluids, such as ascitic or pleural fluid. Critical areas included the ICU, high dependency unit, hematology wards, including the bone marrow transplant unit, and the coronary care unit. Presence of hemodialysis vascular access or a CVC was defined as the presence of either or both devices during the episode of the first positive blood culture for *Candida *species. Steroid use was defined as receipt of high-dose corticosteroids (more than 40 mg of prednisone for more than two weeks) within the previous 30 days or current receipt of corticosteroids, including pulse steroid therapy, during the index admission. Bacteremia was defined as the presence of bloodstream infection caused by a bacterium during the current admission.

## Results

A total of 182 episodes of bloodstream infection caused by *Candida *were identified during the study period from January 2018 to December 2022. Twenty-two patients were excluded from the study, as described in the Materials and Methods section. Therefore, 160 patients were analyzed. The patients were divided into two groups: 58 patients with *C. auris *bloodstream infection, who comprised the *C. auris *group, and 102 patients with bloodstream infection caused by species other than *C. auris*, who comprised the non-*C. auris *group. Among the 102 patients in the non-*C. auris *group, 35 (34.3%) had *C. albicans*, 27 (26.4%) had *C. glabrata*, 15 (14.7%) had *C. parapsilosis*, and 13 (12.7%) had *C. tropicalis*, followed by four cases each of *Candida orthopsilosis *and *Candida dublinensis*, three cases of *C. krusei*, and one case of *Candida lusitaniae*.

The median age in both groups was 61 years. In the *C. auris *group, 35 of 58 patients (60.3%) were admitted to a critical care area, compared with 67 of 102 patients (65.7%) in the non-*C. auris* group. Time to positivity, defined as the time from hospitalization to the first positive blood culture for *Candida*, had a median of 45 days (range, 1-200 days) with an IQR of 74.5 days in the *C. auris *group, compared with a median of 19 days (range, 0-88 days) and an IQR of 9.5 days in the non-*C. auris* group.

The comorbidities observed in both groups are presented in Table 1.

**Table 1:** Comparison of comorbidities in patients with C. auris and non-C. auris bloodstream infections.

Comorbidity	*Candida auris* (n = 58)	Non-*C. auris* (n = 102)	p-value	OR (95% CI)
Diabetes mellitus	27/58 (46.6%)	47/102 (46%)	0.91	1.02 (0.53-1.95)
Hypertension	31/58 (53.4%)	44/102 (43.1%)	0.27	1.51 (0.79-2.89)
Dyslipidemia	23/58 (39.6%)	32/102 (31.4%)	0.37	1.43 (0.73-2.82)
Coronary artery disease	16/58 (27.6%)	26/102 (25.5%)	0.92	1.11 (0.54-2.31)
Obesity	11/58 (18.9%)	11/102 (10.8%)	0.23	1.94 (0.78-4.80)
Chronic kidney disease	20/58 (34.5%)	21/102 (20.6%)	0.08	2.03 (0.99-4.19)
Steroid use	21/58 (36.2%)	21/102 (20.6%)	0.04	2.19 (1.07-4.49)
Inflammatory bowel disease	2/58 (3.45%)	1/102 (0.99%)	0.62	3.55 (0.31-39.9)

The associations studied in the two groups are shown in Table 2. On comparing the two groups, prior colonization with *Candida *species was one of the strongest associations with *C. auris* bloodstream infection, with an OR of 6.71 (95% CI, 3.26-13.80) and a statistically significant p-value of <0.00001.

**Table 2:** Comparison of the associations between C. auris and non-C. auris bloodstream infections. CVC, central venous catheter; TPN, total parenteral nutrition

Association	*Candida auris* (n = 58)	Non-*C. auris* (n = 102)	p-value	OR (95% CI)
Admission to critical area	35 (60.3%)	67 (65.7%)	0.5	0.79 (0.41-1.55)
Prior colonization with *Candida* species	36 (62.1%)	20 (19.6%)	<0.00001	6.71 (3.26-13.80)
Previous health care contact	49 (84.5%)	49 (48%)	<0.00001	5.89 (2.61-13.24)
Solid organ malignancy	13 (22.4%)	15 (14.7%)	0.31	1.68 (0.73-3.82)
Hematological malignancy	7 (12.1%)	6 (5.9%)	0.28	2.20 (0.70-6.88)
Solid organ transplant	2 (3.45%)	2 (1.96%)	0.96	1.79 (0.24-13.02)
Hematopoietic stem cell transplant	4 (6.9%)	2 (1.96%)	0.25	3.70 (0.66-20.88)
Hemodialysis	27 (46.6%)	24 (23.6%)	0.004	2.83 (1.42-5.64)
Presence of CVC	25 (43.1%)	85 (83.3%)	<0.00001	0.15 (0.07-0.32)
Abdominal surgery	10 (17.2%)	22 (21.6%)	0.65	0.76 (0.03-1.74)
TPN use	15 (25.9%)	18 (17.6%)	0.3	1.63 (0.75-3.54)
Urinary catheter use	46 (79.3%)	71 (69.6%)	0.25	1.67 (0.78-3.56)
Presence of bacteremia	36 (62.1%)	50 (49%)	0.15	1.54 (0.79-2.99)

Similarly, previous health care contact, defined as any contact with a health care system within the last 90 days, was significantly associated with *C. auris *bloodstream infection, with an OR of 5.89 (95% CI, 2.61-14.24) and a p-value of <0.00001. Patients on hemodialysis had a significantly higher association with *C. auris* bloodstream infection, with an OR of 2.83 (95% CI, 1.42-5.64; p = 0.004). The presence of CVCs was significantly higher in the non-*C. auris *group compared with the *C. auris *group (83.3% versus 43.1%). The OR of 0.15 suggests that CVC presence was less common in the *C. auris *group than in the non-*C. auris *group. The other factors listed in the table, including the presence of malignancy, transplant recipients, abdominal surgery, TPN, and urinary catheterization, were not statistically significant when comparing the two groups. On comparing the presence of bacteremia (blood culture positivity for bacteria), it was observed in 62% of patients in the *C. auris* group compared with 49% in the non-*C. auris *group. However, this difference was not statistically significant.

Susceptibility testing was performed on 126 isolates, of which 51 were from the *C. auris* group, and 75 were from the non-*C. auris *group. All isolates in the *C. auris *group were resistant to azoles; the drugs tested were fluconazole and voriconazole. Echinocandin susceptibility testing showed that 50 isolates were susceptible to all three echinocandins, namely caspofungin, micafungin, and anidulafungin, whereas one isolate was resistant. For the polyene group, amphotericin B was the tested drug, and 56.9% of isolates were susceptible. Among the non-*C. auris *isolates, 75 of 102 underwent susceptibility testing, and the details are shown in Table 3.

**Table 3:** Comparison of the drug susceptibility patterns between C. auris and non-C. auris bloodstream isolates. SDD, susceptible-dose dependent

Antifungal class	*Candida auris* (n = 58)	Non-*C. auris *(n = 102)
Drug susceptibility available	51	75
Azole susceptibility (includes fluconazole and voriconazole)	0%	61/75 (81.3%) susceptible; 6 resistant (2 *Candida glabrata*, 3 *Candida parapsilosis*, and 1 *Candida krusei)*; five intermediate (*C. glabrata)*; 3 SDD (*C. glabrata*)
Echinocandins (caspofungin, micafungin, and anidulafungin)	50/51 (98.03%) susceptible; 1 resistant	100%
Polyenes (amphotericin B)	29/51 (56.9%) susceptible; 22/51 (43.1%) resistant	100%

Of the total cohort of 182 patients, 78 (42.9%) patients died, and two patients left against medical advice (LAMA). In the *C. auris *group, 30 of 58 patients (51.72%) died, and two patients LAMA. The total number of deaths in the non-*C. auris* group was 48 of 102 patients (47.05%).

## Discussion

*C. auris* is considered a major nosocomial pathogen with substantial implications for health care settings. It is a multidrug-resistant yeast capable of causing hospital outbreaks. It is also well known to cause significant morbidity and mortality among critically ill patients [15]. Since the first reported case of *C. auris* from Oman in 2017, there has been a rise in cases in subsequent years [16].

The aim of our study was to compare the associations, outcomes, and drug susceptibility patterns of patients with *C. auris* and non-*C. auris *bloodstream infections over the five-year study period from January 2018 to December 2022. The most common associations with *C. auris *bloodstream infection were prior colonization with any *Candida *species and previous contact with a health care facility, with ORs of 6.71 (95% CI, 3.26-13.80) and 5.89 (95% CI, 2.61-14.24), respectively. This aligns with global reports that *C. auris *is primarily a health care-associated pathogen with a propensity for nosocomial transmission [18]. The OR for *C. auris *bloodstream infection in patients on hemodialysis was 2.83 (95% CI, 1.42-5.64) compared with non-*C. auris* bloodstream infection.

The presence of CVCs was associated with a higher likelihood of non-*C. auris *bloodstream infection compared with *C. auris *bloodstream infection, with an OR of 0.15 (95% CI, 0.07-0.32). Despite a nonsignificant p-value, *C. auris *was observed in 35 (60.3%) patients admitted to critical areas compared with 67 (65.7%) patients with non-*C. auris *bloodstream infection. This is comparable to the study by Al Maani et al., in which 65.6% of patients with *C. auris *were admitted to the ICU compared with 34.4% who were admitted to medical and surgical noncritical areas [16]. Rudramurthy et al. also demonstrated that *C. auris *candidemia has a predilection toward critically ill patients [19].

In this study, the duration from admission to the first positive blood culture was significantly different between the two groups. The mean time to positivity, as defined in the Methods section, was 47.38 days in *C. auris *infection compared with 12.78 days in non-*C. auris *infection. This observation emphasizes that *C. auris *is a health care-associated infection and that prolonged hospitalization with multiple instrumentation plays a significant role in the development of bloodstream infection compared with non-*C. auris *infection. Studies have demonstrated that, unlike other Candida species, in which the source is endogenous and mainly affects the host through autoinfection, *C. auris *is transmitted through contaminated environments and equipment in health care settings [20]. This finding is also supported by our study, in which previous health care contact was significantly more common among patients with *C. auris *bloodstream infection than among those with non-*C. auris *bloodstream infection (84.5% vs 48%). The majority of *C. auris *cases occur in patients with significant comorbidities and multiple risk factors, including prolonged ICU stays, CVC use, prior antifungal therapy, recent surgery, diabetes mellitus, and exposure to broad-spectrum antibiotics [18]. *C. auris *is considered a notable pathogen due to its ability to form biofilms, which may contribute to therapeutic failure and prolonged colonization.

All isolates in the *C. auris *group demonstrated resistance to azoles, with 100% resistance observed. The azoles tested in our cohort were fluconazole and voriconazole. In contrast, 61 isolates in the non-*C. auris *group were susceptible to azoles. Azole resistance is extremely high and has been demonstrated in multiple studies. Deshkar et al. demonstrated 84% azole resistance among *C. auris *isolates in their study. Other azoles, such as posaconazole and itraconazole, also demonstrated high MICs, indicating resistance [21]. In a recent study, da Silva et al. evaluated the susceptibility of *C. auris *isolates across clades and demonstrated 94% and 96% azole resistance in clades I and III, respectively [22]. For echinocandins, all three agents, namely micafungin, caspofungin, and anidulafungin, were tested in our laboratory. One C. auris isolate was resistant to all three drugs, whereas all non-*C. auris *isolates were susceptible. Other studies have also found that *C. auris *generally exhibits good susceptibility to echinocandins. None of the *C. auris *isolates demonstrated resistance to echinocandins, as reported by Sayeed et al. [19]. In the Kuwaiti study, one isolate was found to be resistant to echinocandins; however, that isolate was identified from a urinary source [23].

When comparing susceptibility to polyenes, for which amphotericin B was the tested drug in our study, 56.9% of *C. auris* isolates were susceptible compared with 100% susceptibility among isolates in the non-*C. auris* group. A study from the same region also demonstrated 23.2% resistance to polyenes among *C. auris *isolates [23]. Similarly, a comparative study conducted in Pakistan reported polyene resistance rates of 3.7% and 0% in the *C. auris *and non-*C. auris *groups, respectively [18]. This contrasted with a study from the Indian subcontinent, where 94% of *C. auris *isolates were susceptible to amphotericin B [21]. Although patients with *C. auris *had prolonged hospitalization compared with patients with non-*C. auris *bloodstream infection, the outcome defined as death during the same admission episode was not significantly different between the two groups. A similar observation was reported in a study conducted in Pakistan by Sayeed et al. [18].

Limitations

This study may be limited by its single-center design in Oman. The comparison of associations between the two groups lacked multivariate analysis, which may represent a limitation of the study. Not all isolates underwent antifungal susceptibility testing, and MICs for each antifungal drug were not available. However, the lack of clinical breakpoints for *C. auris* in existing guidelines poses difficulty in assessing treatment outcomes.

## Conclusions

*C. auris* bloodstream infection was associated with previous health care facility contact, colonization with any *Candida *species, and prolonged hospitalization. Patients undergoing hemodialysis had a higher risk of developing bloodstream infection with *C. auris *compared with non-*C. auris *bloodstream infection. The presence of CVCs increased the risk of bloodstream infection with *Candida *species overall but did not show a direct association with *C. auris *bloodstream infection.

Echinocandins may be considered an empiric choice in patients with suspected *C. auris *bloodstream infection in this region of the world. Although mortality did not differ significantly between the two groups, prolonged hospitalization, previous colonization, and previous health care contact should be considered important associations with* C. auris* bloodstream infection. The use of empiric echinocandins may be lifesaving but can contribute to increased economic and financial burden on the health care system.
